# Adenoviral vector type 26 encoding Zika virus (ZIKV) M-Env antigen induces humoral and cellular immune responses and protects mice and nonhuman primates against ZIKV challenge

**DOI:** 10.1371/journal.pone.0202820

**Published:** 2018-08-24

**Authors:** Freek Cox, Leslie van der Fits, Peter Abbink, Rafael A. Larocca, Ella van Huizen, Eirikur Saeland, Janneke Verhagen, Rebecca Peterson, Jeroen Tolboom, Baerbel Kaufmann, Hanneke Schuitemaker, Dan H. Barouch, Roland Zahn

**Affiliations:** 1 Janssen Vaccines & Prevention B.V., Leiden, The Netherlands; 2 Beth Israel Deaconess Medical Center, Boston, MA, United States of America; Instituto Butantan, BRAZIL

## Abstract

In 2015, there was a large outbreak of Zika virus (ZIKV) in Brazil. Despite its relatively mild impact on healthy adults, ZIKV infection during pregnancy has been associated with severe birth defects. Currently, there is no ZIKV vaccine available, but several vaccine candidates based on the ZIKV membrane (M) and envelope (Env) structural proteins showed promising results in preclinical and clinical studies. Here, the immunogenicity and protective efficacy of a non-replicating adenoviral vector type 26 (Ad26) that encodes the ZIKV M-Env antigens (Ad26.ZIKV.M-Env) was evaluated in mice and non-human primates (NHP). Ad26.ZIKV.M-Env induced strong and durable cellular and humoral immune responses in preclinical models. Humoral responses were characterized by Env-binding and ZIKV neutralizing antibody responses while cellular responses were characterized by ZIKV reactive CD4^+^ and CD8^+^ T cells. Importantly, a single immunization with a very low dose of 4x10^7^ vp of Ad26.ZIKV.M-Env protected mice from ZIKV challenge. In NHP, a single immunization with a typical human dose of 1x10^11^ vp of Ad26.ZIKV.M-Env also induced Env-binding and ZIKV neutralizing antibodies and Env and M specific cellular immune responses that associated with complete protection against viremia from ZIKV challenge as measured in plasma and other body fluids. Together these data provide the rationale to progress the Ad26.ZIKV.M-Env candidate vaccine to clinical testing.

## Introduction

Zika virus (ZIKV) is a mosquito-borne virus that was thought to be restricted to Africa and South-East Asia. From 2013 onwards, it emerged and spread rapidly across the Pacific and into South and Central America and The Caribbean, culminating in a large outbreak in Brazil starting in 2016 [1].

ZIKV infection is generally associated with mild illness. However, the outbreak of ZIKV in 2013 in French Polynesia was associated with approximately 70 cases of Guillain-Barré syndrome [2]. In 2016 the Brazil outbreak was characterized by an association with serious birth defects in infants born to women who became ZIKV infected during pregnancy [3]. Concerns relating to a steep increase in reported cases of microcephaly in babies born to ZIKV positive mothers from 2016 onwards resulted in the World Health Organization declaring a Public Health Emergency of International Concern. In addition to microcephaly, an expanding spectrum of fetal neurological and developmental abnormalities is now associated with intrauterine ZIKV infection, collectively referred to as Congenital Zika syndrome (CZS) [4,5]. To date, little is known about the potential long-term physical and neurobehavioral outcomes for infants born to ZIKV infected mothers with no evident neurological defects at birth. However, findings in NHP indicate that developmental problems are not limited to off-spring born with microcephaly but also seen in off spring with apparently normal development of the brain at birth [6,7].

A ZIKV vaccine would be an effective countermeasure to the rapid transmission and deleterious consequences of ZIKV infection. Several ZIKV vaccine candidates have shown promising preclinical and clinical results. DNA-based and purified inactivated ZIKV vaccine (PIV) candidates that showed protection in mice and NHP against viremia after ZIKV challenge [8–12], have also been recently evaluated in healthy adults [13–16]. Although robust neutralizing antibody titers were induced with DNA-based and PIV vaccine formats, cellular immune responses were only investigated for the DNA-based vaccines where only low levels were achieved.

Replication-incompetent adenovirus vectors, e.g. such derived from the human adenovirus serotype 26 (Ad26), are safe and potent inducers of humoral and cellular immune responses [15,17,18]. It was previously reported that vaccination with a rhesus macaque replication-incompetent adenovirus vector type 52 (RhAd52) encoding the ZIKV membrane protein (M) and the ZIKV envelope antigen (Env) lacking the peptide precursor (pr) (amino acids 216–794 of the polyprotein, RhAd52-M-Env) derived from the ZIKV strain BeH815744 elicited protective immunity against ZIKV challenge in mice and NHP [8,9]. Recombinant chimpanzee adenovirus vector type 7 (AdC7) encoding for identical M and Env antigens (M-Env) also elicited protective immunity against ZIKV challenge and prevented ZIKV-associated testicular damage in mice [11].

Here, we describe immunogenicity and protective efficacy in mice and NHP of a recombinant Ad26-based ZIKV vaccine candidate encoding the M-Env antigens (Ad26.ZIKV.M-Env) produced on the human PER.C6® cell line. The advantage of the modified Ad26 vector used here is that it can be produced to high titers under GMP conditions on human PER.C6® cells at relatively small scale, thereby representing a promising vaccine platform [19,20]. A single immunization with Ad26.ZIKV.M-Env induced ZIKV Env-binding and neutralizing antibody responses as well as strong cellular immune responses in mice and NHP. In addition, full protection against ZIKV challenge was achieved in mice that received a single immunization with a very low dose of Ad26.ZIKV.M-Env and in NHP that received a single immunization with a human dose of Ad26.ZIKV.M-Env.

## Results

### Ad26.ZIKV.M-Env induces durable ZIKV specific humoral immune responses in mice

**Fig 1:**
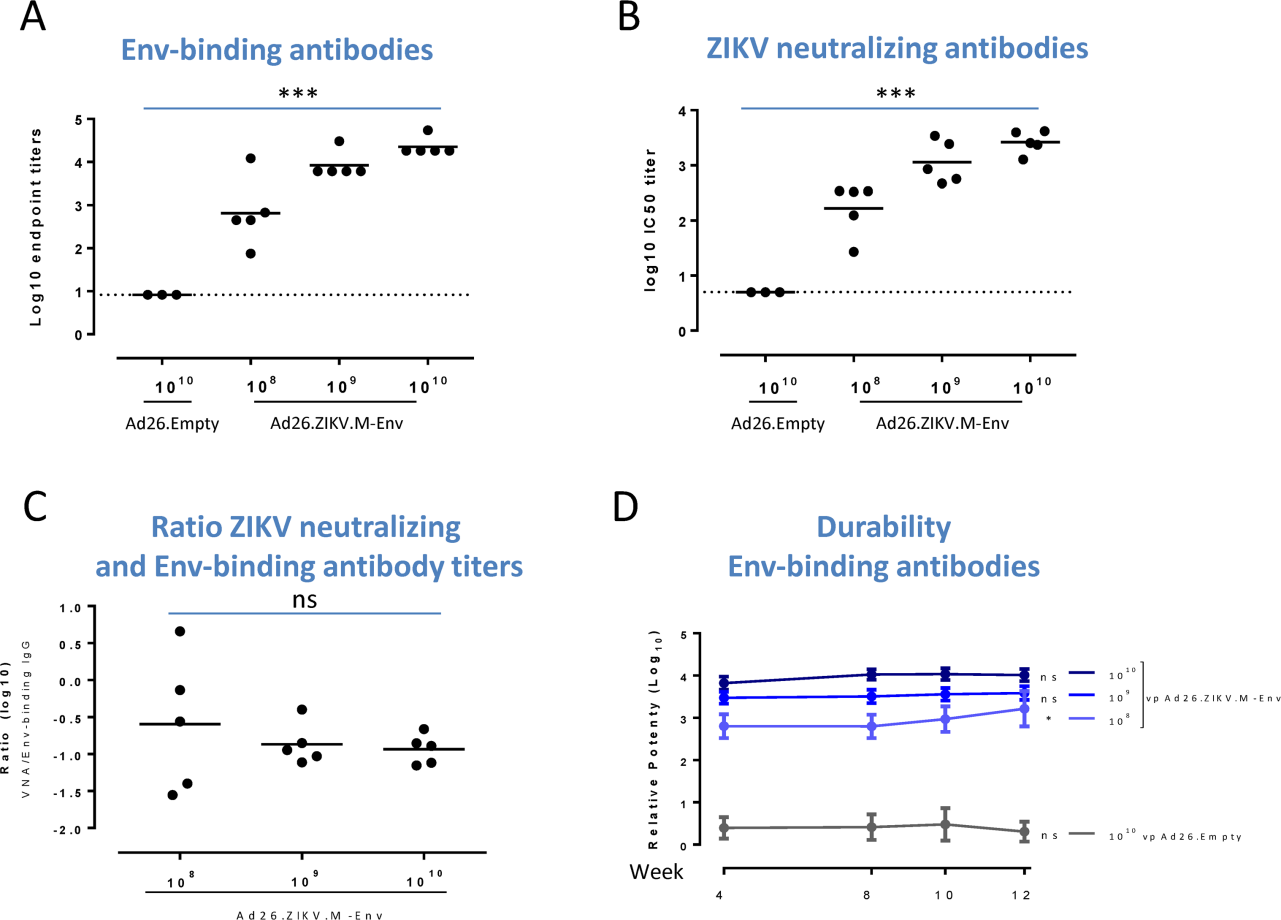
A single immunization with Ad26.ZIKV.M-Env dose dependently induces humoral immune responses in C57BL/6 mice that are maintained for at least 12 weeks. Env-specific binding IgG antibody titers (A) or ZIKV-PR neutralization titers (B) were determined in sera of C57BL/6 mice immunized with Ad26.ZIKV.M-Env (n = 5) or Ad26.Empty control vector (n = 3) at the doses indicated, at 4 weeks post immunization. The titer was determined using a commercially available ELISA kit (Alpha Diagnostics) and expressed as the log10 of the inverse first dilution above 2x background values of naïve sera. ZIKV neutralizing antibody titers were measured by FRNT and are reported as the log10 of the inverse serum dilution that reduce the infectivity of input virus by 50% (IC50). The mean responses per group are indicated with a horizontal line. The dotted line shows the lower limit of detection. (C) The ratio (log10) of ZIKV neutralizing and Env-binding antibody titers. (D) The durability of the Env-specific binding IgG antibody responses in sera of C57BL/6 mice immunized once with 10^8^, 10^9^, or 10^10^ vp Ad26.ZIKV.M-Env or with 10^10^ vp Ad26.Empty (n = 5 per group) was determined at the indicated time points. Env-specific binding antibody titers were measured using an in-house developed ELISA assay. Relative potency (log10) when compared with a ZIKV Env-specific monoclonal antibody ZV-67 [21] was determined, and displayed as mean +/- standard deviation per group. Lines connect the group mean responses per time point. Asterisks indicate statistically significant trend (*p<0.05, **p<0.01 and ***p<0.001) and “ns” indicates no statistical significant trend.

The immunogenicity of Ad26.ZIKV.M-Env was assessed in various mouse strains. Single intramuscular (IM) immunization with 10^8^, 10^9^ or 10^10^ virus particles (vp) of Ad26.ZIKV.M-Env in C57BL/6 mice resulted in a dose-dependent induction (p<0.001, trend analysis) of Env-specific binding 4 weeks post immunization (Fig 1A). Sera were additionally assayed for the presence of ZIKV neutralizing antibody responses by Focus Reduction Neutralization test (FRNT) using the ZIKV-PR strain. A dose-dependent induction (p<0.001, trend analysis) of ZIKV neutralizing titers in serum, 4 weeks after immunization was shown (Fig 1B). The ratio between Env-binding and ZIKV neutralizing antibody responses were (Fig 1C). The dose-dependent induction of Env-specific binding and ZIKV neutralizing antibody titers as well as their correlation were confirmed in Balb/c and SJL mice (parts A-F of S1 Fig).

The durability of the Env-binding antibody response was evaluated in C57BL/6 mice immunized once with 10^8^, 10^9^ or 10^10^ vp of Ad26.ZIKV.M-Env. Env-binding antibodies were measured in an in-house developed ELISA that highly correlates (r^2^ = 0.979) with the commercially available and widely used ZIKV Env ELISA (Alpha Diagnostics) (part G of S1 Fig). A dose-dependent induction of Env-specific binding antibodies was observed from 4 weeks after immunization that did not decline over 12 weeks. Notable, the Env-specific antibody response marginally increased in time in the group that received the lowest Ad26.ZIKV.M-Env dose (; p = 0.019, p = 1.000, p = 0.446 for 10^8^, 10^9^ or 10^10^ vp, respectively, trend analysis of responses over time) (Fig 1D).

### Ad26.ZIKV.M-Env induces durable cellular immune responses directed against both encoded ZIKV antigens in mice

**Fig 2:**
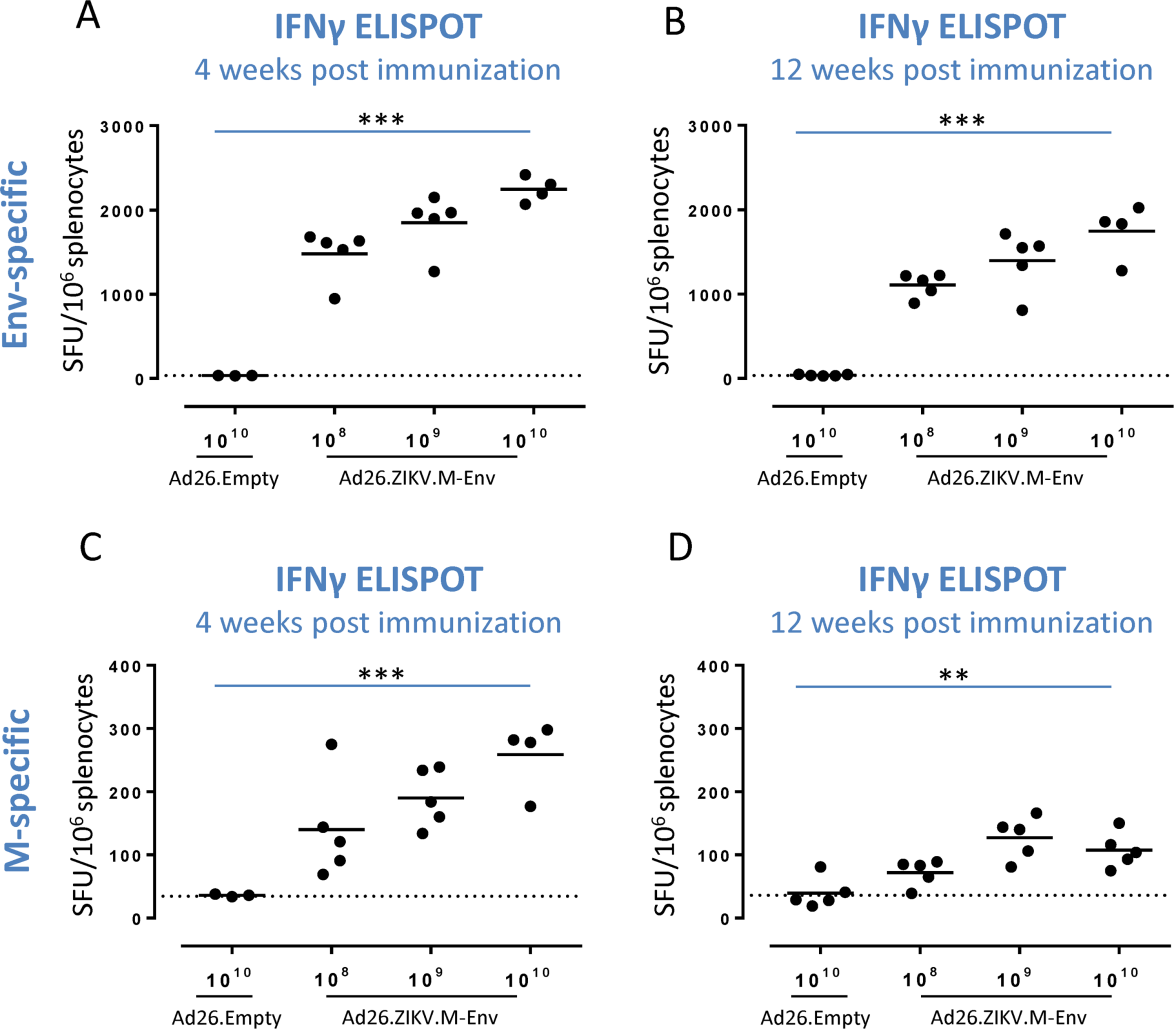
Ad26.ZIKV.M-Env dose dependently induces durable ZIKV-specific cellular responses after a single immunization in C57BL/6 mice. Env and M-specific IFNγ responses were determined by ELISPOT in splenocytes from C57BL/6 mice immunized with Ad26.ZIKV.M-Env (n = 5) or Ad26.Empty (n = 3 or 5) at the doses indicated, at 4 or 12 weeks post immunization. Splenocytes were stimulated overnight with Env-specific (A-B) or M specific (C-D) peptide pools. The number of IFNγ spot forming units (SFU) per 10^6^ splenocytes is shown. The geometric mean response per group is indicated with a horizontal line. The dotted lines indicate the background of the assays. Asterisks indicate statistically significant trend (*p<0.05, **p<0.01 and ***p<0.001) and “ns” indicates no statistical significant trend.

Cellular responses directed to Env and M-derived peptide pools were measured by IFNγ ELISPOT assay in splenocytes of C57BL/6 mice, isolated at 4 or 12 weeks after single immunization with 10^8^, 10^9^ or 10^10^ vp of Ad26.ZIKV.M-Env. A dose-dependent induction of Env-specific IFNγ response (p<0.001, trend analysis) was detected at 4 weeks post-immunization that was still present at week 12 post immunization (Fig 2A and 2B). Ad26.ZIKV.M-Env also induced a dose-dependent (p<0.001, trend analysis) cellular responses to M-derived peptides 4 weeks after prime immunization that were maintained up to 12 weeks after immunization (Fig 2C and 2D).

### Ad26.ZIKV.M-Env immunization induces ZIKV-reactive CD4^+^ and CD8^+^ T cell in mice

**Fig 3:**
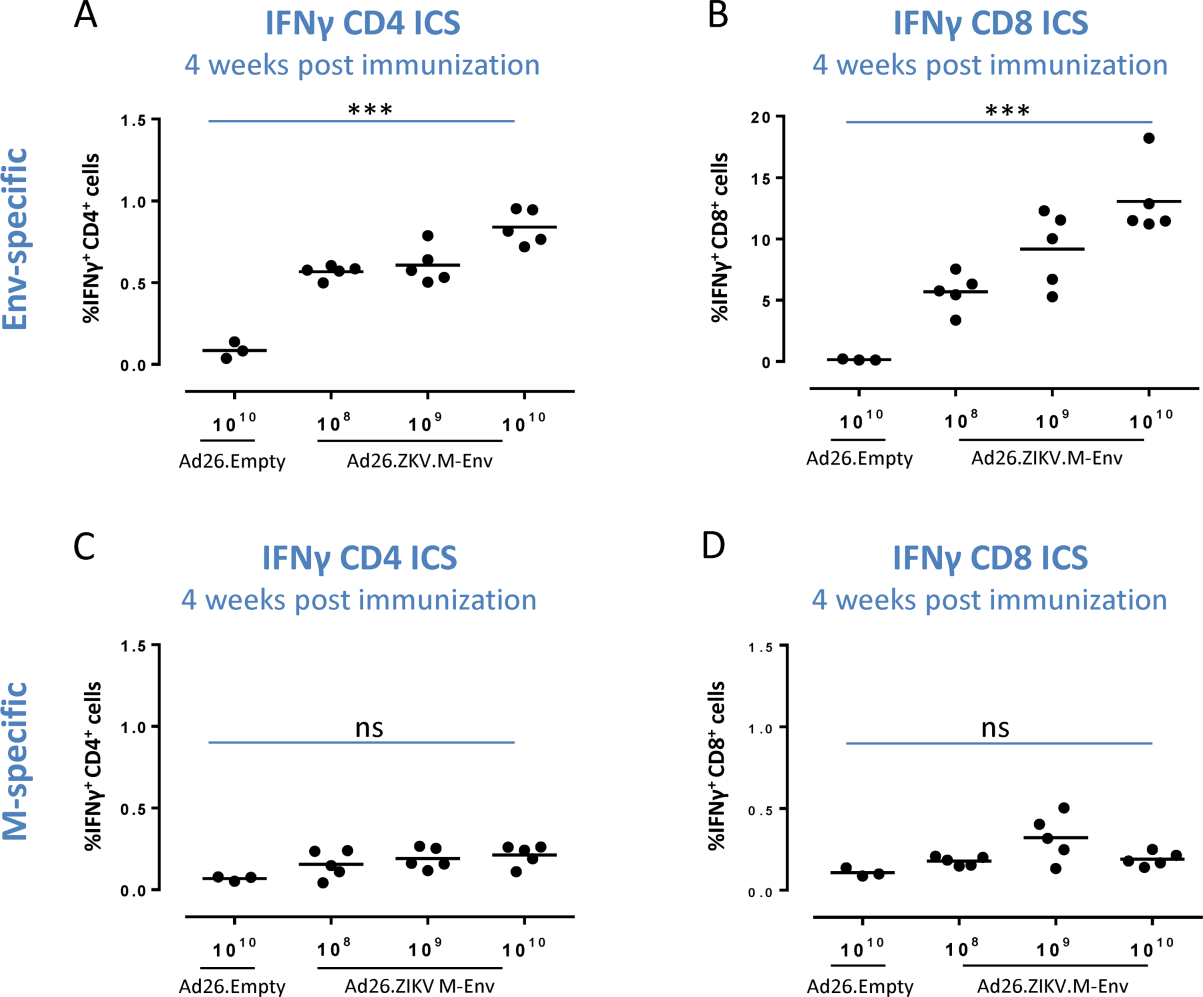
A single immunization with Ad26.ZIKV.M-Env dose dependently induces ZIKV-specific CD4^+^ and CD8^+^ reactive T cells in C57BL/6 mice. Env and M-specific IFNγ responses were determined by ICS in splenocytes from C57BL/6 mice immunized with Ad26.ZIKV.M-Env (n = 5) or Ad26.Empty control vector (n = 3) at the doses indicated, at 4 weeks post immunization. Splenocytes were stimulated overnight with Env-specific (A-B) or M specific (C-D) peptide pools. The percentage of CD3^+^CD4^+^ and CD3^+^CD8^+^ splenocytes producing IFNγ is depicted. The geometric mean response per group is indicated with a horizontal line. Asterisks indicate statistically significant trend (*p<0.05, **p<0.01 and ***p<0.001) and “ns” indicates no statistical significant trend.

To characterize cytokine producing T-cell populations more closely, intracellular cytokine staining (ICS) followed by FACS analysis was performed using splenocytes of C57BL/6 mice isolated 4 weeks after single immunization with 10^8^, 10^9^ or 10^10^ vp of Ad26.ZIKV.M-Env. This ICS analysis showed that the Env-directed cellular responses were dominated by IFNγ secreting CD8^+^ T cells, with at the highest vaccine dose up to 15% of the CD8^+^ cells being positive for IFNγ (Fig 3B). However, Ad26.ZIKV.M-Env also induced dose-dependent Env-specific, IFNγ secreting CD4^+^ T-cell responses after a single immunization, although to a lower extent (up to 0.9% at the highest vaccine dose) (Fig 3A). The percentage of Env-specific CD4^+^ and CD8^+^ T-cells secreting TNFα were in the same range as Env-specific IFNγ responses, while the IL-2 responses were lower, but clearly above background levels of Ad26-Empty immunized mice (parts A-D of S2 Fig). ICS data showed that cellular responses to M were present within CD8^+^ and CD4^+^ subpopulations, but the responses were lower when compared to Env directed responses (Fig 3C and 3D and parts E-H of S2 Fig).

Cellular responses induced by Ad26.ZIKV.M-Env to the Env-derived peptide pool were also measured in Balb/c and SJL mice, and response level varied between the different mouse strains (parts A-B of S3 Fig). In Balb/c mice the cellular responses directed to M were low to non-detectable, while in SJL mice immunization with Ad26.ZIKV.M-Env induced detectable cellular responses to M (parts C-D of S3 Fig). These differences demonstrate that the choice of animal model can strongly influence immunological outcomes and subsequent interpretation.

### A second immunization with Ad26.ZIKV.M-Env boosts CD8^+^ T-cell responses

**Fig 4:**
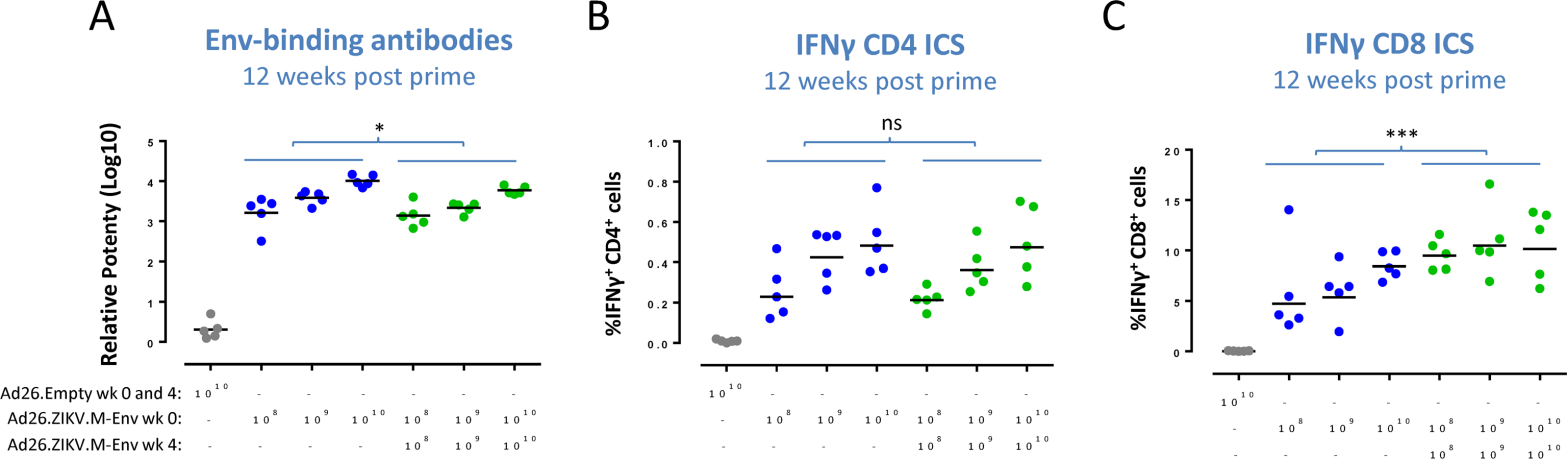
Env-specific CD8^+^ T-cell responses are increased, but Env-specific binding antibody and CD4^+^ T-cell responses do not increase after boost immunization. Env-specific binding IgG antibody titers (A) were determined in sera of C57BL/6 mice prime or prime boost immunized with Ad26.ZIKV.M-Env (n = 5) at the doses indicated, at 12 weeks after prime immunization by using an in-house developed ELISA assay. Dots represent individual responses of the relative potency (log10) compared to a ZIKV Env-specific monoclonal antibody ZV-67, and the mean per group is indicated with a horizontal line. Env-specific splenic CD4^+^ (B) and CD8^+^ T cells (C) secreting IFNγ were determined after overnight stimulation with Env-specific peptide pools. Dots represent percentage IFNγ^+^ T cells of CD3^+^CD4^+^ or CD3^+^CD8^+^ gated cells, and geometric mean responses are indicated with a horizontal line. Blue (prime only) and green (prime-boost). Asterisks indicate statistically significant trend (*p<0.05, **p<0.01 and ***p<0.001) and “ns” indicates no statistical significant trend.

To determine whether the humoral and cellular immune responses induced by Ad26.ZIKV.M-Env could be boosted by a second homologous immunization with Ad26.ZIKV.M-Env, C57BL/6 mice were immunized with 10^8^, 10^9^, or 10^10^ vp Ad26.ZIKV.M-Env in a homologous prime-boost regimen with a 4-week prime-boost interval. In general, relative stable titers were achieved over time by both regimens and a comparison was made between a prime only and prime-boost regimen. Env-specific antibody titers were similar for prime-only and prime-boost groups (part A of S3 Fig), and with no significant difference between the groups at week 8 and 10 post-prime immunization in a across dose comparison (part B-C of S3 Fig), and were marginally, but significantly lower at 12 weeks in the groups that received a boost immunization as compared to the groups that received a prime only immunization (across-dose difference testing, p = 0.017) (Fig 4A).

At 12 weeks post prime immunization, dose-dependent Env-specific IFNγ secreting CD4^+^ and CD8^+^ T-cell responses in splenocytes were detected in all groups that received one or two immunizations with Ad26.ZIKV.M-Env (Fig 4B and 4C). CD8^+^, but not CD4^+^, T-cell responses were significantly higher after prime-boost immunization with Ad26.ZIKV.M-Env compared with the responses in animals that received a single immunization, when analyzed in an across-dose comparison (difference testing p<0.001) (Fig 4C).

### Ad26.ZIKV.M-Env immunization protects mice against ZIKV viremia after ZIKV challenge

A single immunization with Ad26.ZIKV.M-Env induced ZIKV Env-binding and neutralizing antibody responses as well as Env and M specific cellular immune responses in various mouse strains, including Balb/c mice. Balb/c mice were used to evaluate the protective efficacy of a single immunization with Ad26.ZIKV.M-Env at three different doses (4×10^7^, 2×10^8^ or 1×10^9^ vp). The dose range was based on previous immunogenicity results that showed induction of Env-binding antibodies in Balb/c mice that received 10^9^ and 10^8^ vp, but not in mice that received 10^7^ Ad26.ZIKV.M-Env (part A-B of S1 Fig). Ad26.Empty at a ‘highest dose equivalent’ to Ad26.ZIKV.M-Env vaccine was included as a sham control. Animals were challenged 4 weeks post immunization with ZIKV strain Brazil-ZKV2015 (ZIKV-BR) via the intravenous route.

**Fig 5:**
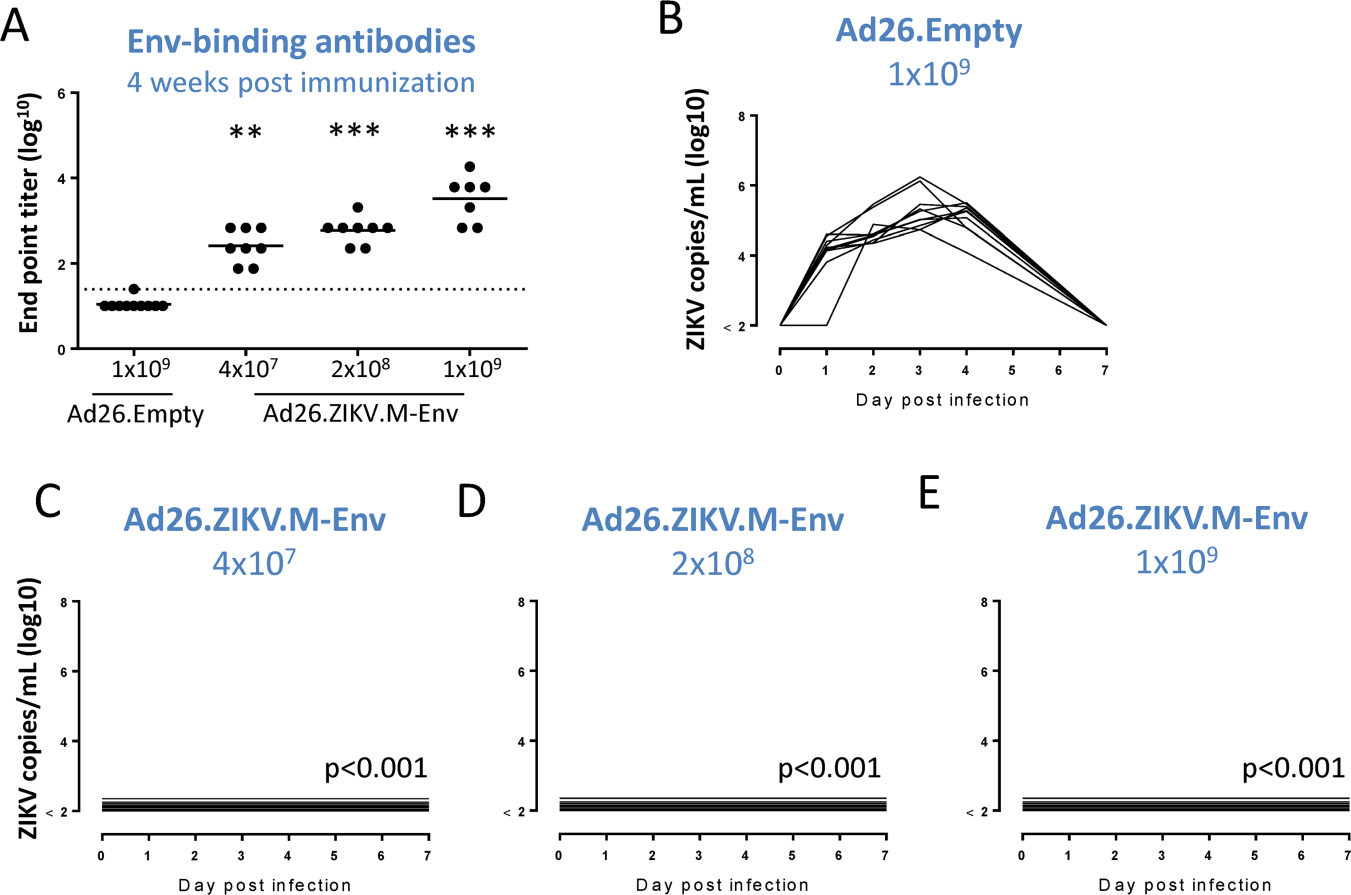
Ad26.ZIKV.M-Env induces protective efficacy against ZIKV-BR challenge in Balb/c mice. Animals (n = 8 or 10 per group) were immunized with Ad26.ZIKV.M-Env or Ad26.Empty control vector as indicated. Env-specific binding IgG antibody responses in sera were determined 4 weeks post immunization (A) using a commercially available ELISA kit (Alpha Diagnostics) and expressed as the log10 of the inverse first dilution above 2x background values of naïve sera. The mean responses per group are indicated with a horizontal line. The dotted line shows the lower limit of detection. Four weeks post-immunization animals were challenged via the intravenous route with 10^2^ pfu ZIKV-BR. Serum was obtained at day 1–4 and 7 post challenge to determine viral loads by RT-PCR, depicted as log^10^ ZIKV copies/mL serum (B-E). The limit of detection of this assay was <100 copies/mL serum. Asterisks indicate statistically significant differences (*p<0.05, **p<0.01 and ***p<0.001) and “ns” indicates no statistical significant difference as compared to the sham control group.

Immunization with the various doses of Ad26.ZIKV.M-Env resulted in a dose dependent induction of Env-specific binding antibody titers with seroconversions observed in all animals that received Ad26.ZIKV.M-Env (p≤0.001 for all doses compared to Ad26.Empty) (Fig 5A). All animals that received Ad26.Empty showed detectable ZIKV RNA loads in the serum, with peak titer at day 3 to 4 post-challenge (Fig 5B). In contrast, mice immunized with 4×10^7^, 2×10^8^ or 1×10^9^ vp of Ad26.ZIKV.M-Env were all protected against viremia after challenge with ZIKV-BR, as evidenced by undetectable viral RNA loads in the isolated serum samples (p<0.001 for all doses compared to Ad26.Empty) (Fig 5C–5E).

### Ad26.ZIKV.M-Env is immunogenic and provides full protection in rhesus macaques

Immunogenicity and protective efficacy of Ad26.ZIKV.M-Env was further assessed in NHP (rhesus macaques). The animals received a single immunization with a typical human dose of 10^11^ vp Ad26.ZIKV.M-Env or formulation buffer as sham control and were challenged subcutaneously with ZIKV-BR 4 weeks post-immunization.

Limited safety assessments were performed post-immunization and included the assessment of injection site reactions (using Draize score; part A of S1 Table), clinical signs (cage side observations), hematology parameters, as well as body weight and body temperature (S2 Table). The administration of a single full human Ad26.ZIKV.M-Env dose of 1×10^11^ vp was well tolerated in all study animals and no vaccine-related adverse effects were noted.

**Fig 6:**
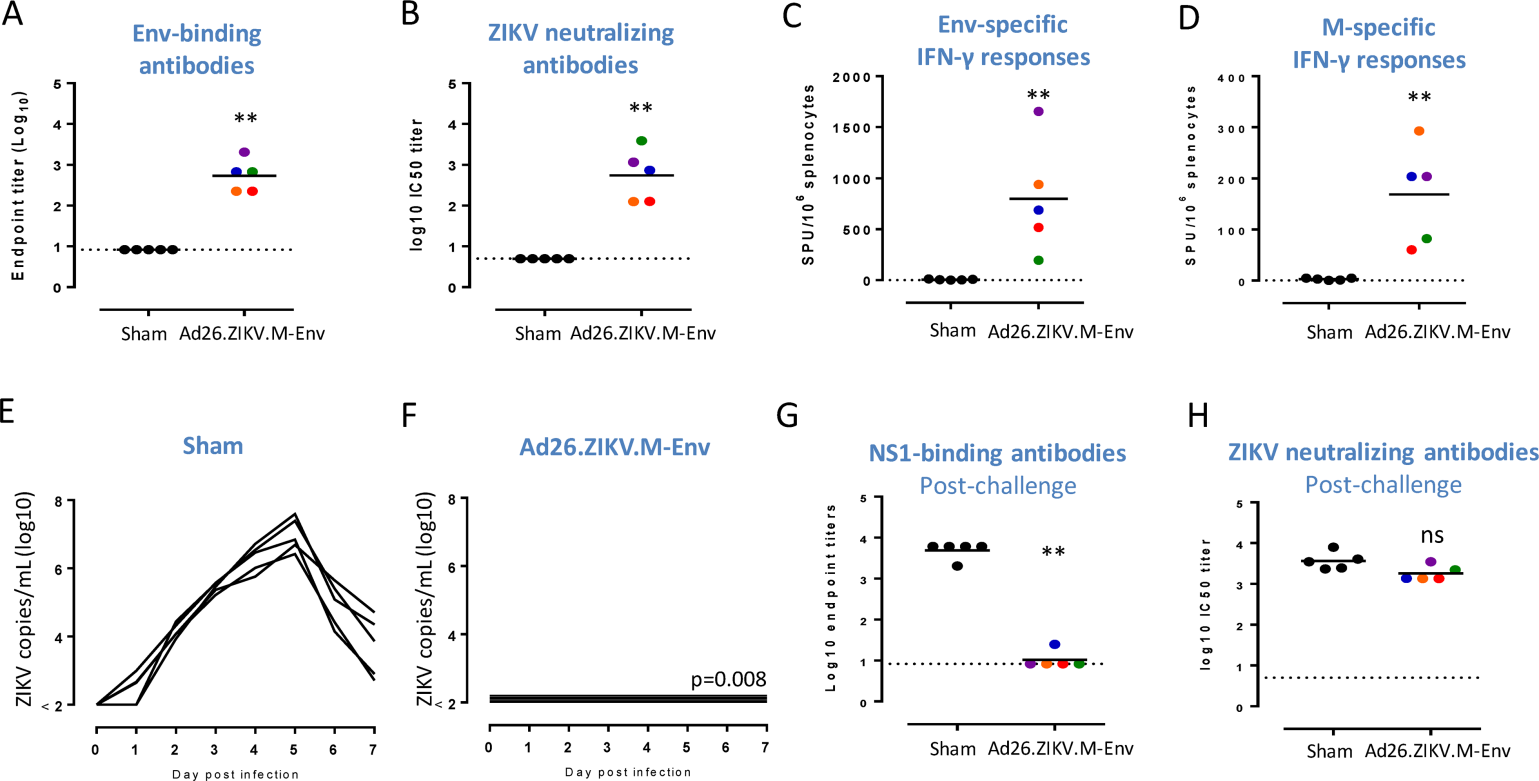
A single immunization with Ad26.ZIKV.M-Env induces humoral and cellular immune responses in NHP and completely protects NHP from ZIKV viremia. Humoral and cellular responses were measured in NHP (n = 5 per group) immunized with 10^11^ vp Ad26.ZIKV.M-Env or formulation buffer (Sham), at 4 weeks post-immunization (pre-challenge). (A) Env-specific binding IgG antibody responses in sera were determined by ELISA (Alpha Diagnostics) and expressed as the log10 of the inverse first dilution above 5x the background value of naïve samples. (B) ZIKV-PR neutralization titers were measured by FRNT assay and reported as the log10 of the inverse serum dilution that reduce the number of input virus by 50% (IC50). (C and D) IFN-γ ELISpot responses were measured in PBMCs overnight stimulated with Env- (C) or M-specific (D) peptide pools and displayed as IFN-γ spot forming units (SFU) per 10^6^ PBMCs. (E-F) Viremia was determined after a subcutaneous challenge with 10^3^ pfu ZIKV- 4 weeks post-immunization by RT-PCR in plasma obtained pre-challenge and 7 consecutive days after challenge and depicted as log10 ZIKV copies/mL plasma. At 4 weeks post-challenge, NS1 binding antibody titers (G), and ZIVK-PR neutralization titers (H) were determined by ELISA (Alpha Diagnostics), and FRNT, and expressed as the log10 of the inverse first dilution above 2x the background value of naïve sera and the log10 of the inverse serum dilution that reduce the number of input virus by 50% (IC50), respectively. The mean (A-B and G-H) or geometric mean (D-E) response per group is indicated with a horizontal line. Colored dots represent individual animals. The dotted lines show the lower limit of detection. Asterisks indicate statistically significant differences (*p<0.05, **p<0.01 and ***p<0.001) and “ns” indicates no statistical significant difference as compared to the sham control group.

Ad26.ZIKV.M-Env induced humoral immune responses in NHP, as shown by the induction of Env-binding antibody titers (Fig 6A), and ZIKV-PR neutralizing antibody titers (Fig 6B), that were significantly higher compared with the response in sham injected animals (p = 0.008 for both comparisons). In addition, there was a significant induction of Env- and M- specific IFNγ secreting PBMCs 4 weeks post immunization with Ad26.ZIKV.M-Env, compared with the response in sham injected animals (p = 0.008) (Fig 6C and 6D).

All sham-injected NHP showed viral loads in the plasma after ZIKV-BR challenge (Fig 6E). In contrast, NHP immunized with Ad26.ZIKV.M-Env were all protected against viremia after challenge with ZIKV-BR, as evidenced by undetectable viral ZIKV RNA loads in plasma samples from these animals (p = 0.008 when compared with sham injected animals) (Fig 6F). In addition, the ZIKV viral loads in cerebrospinal fluid (CSF), urine, and in saliva after ZIKV-BR challenge were measured at days 3 and 7 post challenge. Whereas ZIKV RNA was detectable in CSF samples of all sham-immunized animals, no virus was detected in CSF samples of Ad26.ZIKV.M-Env vaccinated animals (p = 0.008, compared with sham injected animals) (parts A-B of S5 Fig). Viral RNA was also detected in some urine (1 out of 5 animals) and saliva samples (3 out of 5 animals) of sham-injected animals, whereas no viral RNA was measured in any of these body fluids from Ad26.ZIKV.M-Env immunized animals (parts A-F of S5 Fig).

Four weeks after challenge, non-structural protein 1 (NS1)-binding and ZIKV neutralizing antibody responses were assessed as an indicator if sterile protection was achieved. Sham injected, ZIKV-BR challenged animals developed NS1-binding antibody titers as response to replicating ZIKV challenge virus, while NS1 binding antibody titers were low to undetectable in Ad26.ZIKV.M-Env immunized animals that were protected from ZIKV viremia (p<0.001 comparing Ad26.ZIKV.M-Env and sham) (Fig 6G). As expected, sham-immunized animals developed ZIKV neutralizing titers in serum upon ZIKV challenge. ZIKV neutralizing titers of animals that were immunized with Ad26.ZIKV.M-Env, and protected from ZIKV viremia, remained essentially unchanged upon ZIKV challenge (mean titers of 2.73±0.40 pre-challenge, and 3.24±0.19 post-challenge; p = 0.56) (Fig 6H).

## Discussion

Here we show that Ad26 encoding for ZIKV M and Env is highly immunogenic in several preclinical models. Ad26.ZIKV.M-Env induced strong humoral immune responses in mice and NHP that were characterized by antibodies capable of binding the ZIKV Env protein, and in vitro neutralization of the vaccine-heterologous ZIKV-PR strain. Our vaccine also induced cellular immune responses in both mice and NHP. In Balb/c mice a single low dose immunization with just 4×10^7^ vp of Ad26.ZIKV.M-Env was sufficient to protect against viremia induced by intravenously injected ZIKV-BR. In NHP, a single immunization with a typical human dose (1×10^11^ vp) of Ad26.ZIKV.M-Env provided complete protection against viremia after subcutaneous ZIKV challenge. These results are in line with, and further elaborate on, results obtained previously with a RhAd52 vector encoding for the same M-Env transgene [8,9,22]. RhAd52-M-Env was shown to elicit Env-binding, ZIKV neutralizing antibody and cellular immune responses and providing protection against ZIKV challenge in mice dosed with 10^9^ vp and NHP dosed with 10^11^ vp 4 weeks after immunization [8,9]. The protection in these animal models correlated with Env-specific binding antibody and ZIKV neutralizing antibody responses assessed by serum transfer studies [9,22].

The immune durability data presented here demonstrate that Env-directed antibody and cellular immune responses induced after a single immunization with Ad26.ZIKV.M-Env are maintained for at least 12 weeks in mice. The second immunization only boosted the CD8^+^ T-cell responses and not the CD4^+^ T-cell response or the Env-directed antibody response. This is in alignment withour experience that homologous prime-boost regimens with Ad26-based vaccines rarely increases the antibody response in mice, while in humans the prime response can be boosted by an additional immunization [18].

Our durability results are in accordance with data obtained with Ad26 and RhAd52-based ZIKV vaccine candidates given at high dose encoding for the M-Env antigen, which showed that ZIKV neutralizing antibodies resulted in protection in mice that were challenged 20 weeks after immunization. The durability of protective immune responses induced by RhAd52-M-Env was confirmed in NHP, where ZIKV challenge of RhAd52-M-Env immunized animals at 52 weeks after immunization resulted in complete protection in 100% of the animals. In contrast, a ZIKV DNA vaccine and purified inactivated virus (PIV) provided NHP with complete protection at peak immunity, but did not confer complete protection against challenge at 52 weeks post-immunization [22].

Longevity of protection has not been assessed in humans yet, however the PIV ZIKV vaccine adjuvanted with alum and two DNA-based vaccines have been evaluated in phase 1 clinical trials. Two immunizations with PIV 4 weeks apart resulted in neutralizing antibody titers in nearly all subjects (92%) at 2–4 weeks after the boost. Human-to-mouse serum transfer studies demonstrated that the induced antibody responses provided partial to complete protection against viremia in ZIKV challenged mice [13]. The response induced by two different DNA-based ZIKV candidate vaccines showed different outcomes depending on the candidate and regimen. The most promising candidate, VRC5283, containing the wild-type prM-Env, yielded in 100% of subjects (14/14) detectable neutralizing antibody responses with the highest geometric mean titers after 3 administrations with a needle-free device with a split-dose (into two arms) [14]. Another DNA-based vaccine encoding for prM-Env showed induction of Env-binding antibodies in 100% of the subjects, while only up to 63% developed ZIKV neutralizing antibody responses and moderate T-cell responses after 3 immunizations. Immune serum transferred to AG129 IFNα knockout mice provided protection against lethal ZIKV challenge in 103 out of 112 recipients (93%) [16].

It is considered critical for a ZIKV vaccine to induce durable protective immunity. RhAd52-M-Env and Ad26-M-Env both elicit durable protective immunity in mice and RhAd52-M-Env also elicit durable immunity in NHP [22]. Immune responses induced with other Ad26-based vaccine candidates that we already tested in humans are also durable [13–15] and supportive of moving the Ad26.ZIKV.M-Env ZIKV candidate vaccine into clinical development.

Although neutralizing antibodies have been identified as the primary mechanistic correlate of protection for ZIKV vaccine candidates in NHP and by human-to-mouse serum transfer studies, there is growing evidence that T cells also contribute to protective immunity. It has been shown that mice with impaired CD8^+^ T-cell compartments are more susceptible to ZIKV challenge [23] and that mice that were infused with DENV-immune CD8^+^ T cells showed reduced viral burden after ZIKV challenge [24]. These data show that T cells can contribute to protection in the absence of protective antibodies. Here, we show that robust cellular responses were induced in mice and NHP. In mice, the cellular responses were predominantly characterized by CD8^+^ T cells secreting IFNγ or TNFα, although relatively high levels of CD4^+^ responding T cells were also induced upon immunization and that these cellular responses were maintained for at least 12 weeks.

During Phase 1 evaluation of DNA-based ZIKV vaccine candidates limited but significant CD4^+^ and CD8^+^ T-cell responses were induced [14,16]. In NHP, two immunizations with alum adjuvanted PIV has been shown to induce cellular responses in the same range as a single immunization with RhAd52-M-Env [8]. The level of cellular immune responses induced by alum adjuvanted PIV in humans is currently not yet publicly available. However, PIV and DNA-based vaccine candidates are likely not the ideal vaccine platforms to induce high and durable cellular immune responses in humans. In contrast, Ad26-based vaccine candidates have demonstrated to be excellent inducers of cellular responses and belong to one of the few vaccine platforms able to induce durable CD8^+^ T cells in humans [15,17,18].

Although human ZIKV disease is relatively mild in healthy adults, ZIKV can cause severe birth defects in fetuses of ZIKV positive pregnant women that are associated with dysfunction of ZIKV infected neuroprogenitor cells [25]. There are strong indications that ZIKV efficiently replicates in fetal tissue, forming a viral reservoir that could be the reason for the observed prolonged viremia (>14 days) during pregnancy compared with non-pregnant individuals (7–10 days) [26,27]. Several preclinical models have been used to study vaccine efficacy during pregnancy. It has been shown that transferred ZIKV specific monoclonal antibodies or immune responses induced by mRNA-based or live attenuated ZIKV candidate vaccines were able to prevent fetal demise in mice [28–30]. Studying infection in pregnant mice might yield valuable information regarding the ability of the vaccine to prevent birth defects. However, NHP more closely resemble human gestation, including placentation, brain structure and neurodevelopment and are considered more appropriate for studying infection in pregnancy [31]. Recently several studies investigated the impact of ZIKV infection during pregnancy in NHP and showed that neurological pathology occurred in brains of fetuses of infected pregnant NHP, particularly when ZIKV inoculation occurred early in pregnancy [7,32–35]. These models could be suitable to test intervention strategies including vaccination to prevent fetal infection. Since ZIKV seems to replicate more efficiently during pregnancy, it is likely necessary to achieve near sterile protection by vaccination to prevent congenital Zika syndrome. It remains to be elucidated whether the observed protection induced by Ad26.ZIKV.M-Env in NHPs directly translates into prevention of congenital Zika syndrome or that Ad26.ZIKV.M-Env might indirectly contribute to prevention of congenital Zika syndrome by heard immunity.

Protective efficacy of Ad26.ZIKV.M-Env against ZIKV viremia in NHP resulted in near undetectable NS1-binding antibody titers four weeks after ZIKV challenge in Ad26.ZIKV.M-Env immunized animals. The NS1 protein is not associated with ZIKV viral particles nor is it one of the components of Ad26.ZIKV.M-Env, and is thus only presented to the immune system by infected cells [36]. Therefore, the NS1 ELISA results suggest near sterile protection was achieved in NHP that received a single Ad26.ZIKV.M-Env immunization. In contrast, sham injected animals displayed viral load after ZIKV challenge and developed robust NS1 titers.

The robust induction of NS1-binding antibodies after ZIKV exposure in non-protected NHPs, and the near-absence of NS1-binding antibody titers in ZIKV exposed protected animals suggests that NS1-binding antibody responses could be used as surrogate clinical readout for ZIKV infection in efficacy studies. This would have a significant benefit over molecular-based virology readouts with a narrow detection window for ZIKV RNA in ZIKV infected humans [37,38]. It has already been shown that serodiagnostics of ZIKV infections based on NS1-reactive antibodies is highly accurate and avoids false positive detection of ZIKV as a result of cross-reactive antibodies induced by other Flaviviridae such as Dengue virus (DENV) [39,40].

In summary, a single immunization with Ad26.ZIKV.M-Env induces Env-binding and ZIKV neutralizing antibody responses and cellular immune responses including CD4^+^ and CD8^+^ T-cell populations that translate to full protection against ZIKV viremia in mice and potentially sterile protection in NHPs that is likely to be durable. Together these data have prompted phase 1 clinical testing of our Ad26.ZIKV.M-Env vaccine candidate (Ad26.ZIKV.001) (NCT03356561).

## Materials and methods

### Ethical statement

All mouse studies were compliant with the Dutch Law and Guidelines on the Protection of Experimental Animals by the Council of the European Committee (EU Dir. 86/609) and approved by an internal independent expert committee (Animal welfare body) or approved by the Beth Israel Deaconess Medical Center (Boston, MA, USA) Institutional Animal Care and Use Committee (IACUC) (protocol number 005–2015). Female C57BL/6, SJL or Balb/c mice were housed at Innoser Laboratories B.V. (Leiden, The Netherlands), Janssen Vaccine & Prevention B.V. (Leiden, The Netherlands) or at Beth Israel Deaconess Medical Center (Boston, MA, USA) under specified pathogen free conditions.

Mice were monitored daily after immunization or challenge. No clinical signs (score 0) were observed during the in vivo phase of the studies. Clinical scores were defined as: 0 = no clinical signs, 1 = rough coat, 2 = rough coat, less reactive, passive during handling, 3 = rough coat, rolled up, labored breathing, passive during handling, 4 = rough coat, rolled up, labored breathing, unresponsive. One SJL mouse of the group that received 1x10^9^ vp of Ad26.ZIKV.M-Env of the immunogenicity experiment and one Balb/c mouse of in the group that received 1x10^9^ vp of Ad26.ZIKV.M-Env of the challenge experiment died during the blood collection due to technical failures. At the end of the studies, mice of the immunogenicity studies were euthanized by cervical dislocation under isoflurane anesthesia and mice included in the challenge study were euthanized by CO_2_ exposure after isoflurane anesthesia. A total of 51 C57BL/6, 18 SJL and 18 Balb/c mice were used in the immunogenicity experiments and 34 Balb/c mice were used in the challenge experiment.

The NHP research protocols was approved by the BIOQUAL’s IACUC (protocol number 17–017) and were in compliance with the Animal Welfare Act, Public Health Service Policy on humane care and use of laboratory animals, and other federal statutes and regulations relating to animals and experiments involving animals. The studies were conducted in one of BIOQUAL’s AAALAC (International Association for the Assessment and Accreditation of Laboratory Animal Care) accredited facilities. Ten outbred, Indian-origin, macaca mulatta (rhesus macaque) between the ages of 3–8 years were purchased from PrimGen, Inc. (Hines, IL). NHPs were housed singly during the study period to avoid transmission of virus between animals in a 2- or 4-pack cage system according to AAALAC and United States Department of Agriculture (USDA) standards. Each cage had a floor area of 4.3–6.0 sq. ft and a height of at least 30 inches. During the course of the study, animals were provided structural (perch), inanimate (manipulable toys), and food enrichment. Food enrichment was provided at 5–7 days per week and consisted of portions of fruits and vegetables. Additional enrichment (foraging tasks, etc) were provided at least once a week. Cage side animal health observations were performed twice daily and evaluated according to a standardized scoring rubric. No clinical signs or abnormalities were observed during the in vivo phase of the study. For all biological procedures, the animals were anesthetized intramuscularly with 10 mg/kg ketamine. Euthanasia was performed in accordance with the recommended method of the Panel on Euthanasia of the American Veterinary Medical Association. Animals were sedated prior to administration of an overdose of pentobarbital sodium via the intravenous route.

### Vaccine, immunization and challenge

Ad26.ZIKV.M-Env is a monovalent recombinant Ad26-based ZIKV vaccine candidate that encodes for ZIKV membrane (M) lacking the peptide precursor (pr), and envelope (Env) antigens (amino acids 216–794 of the polyprotein) derived from the ZIKV strain BeH815744 [41]. The vaccine was produced on the human PER.C6® cell line and purified and characterized as described previously [19]. Ad26 particle concentrations were determined by optical density at 260nm and viral infectivity by TCID50 assay. All vaccine preparations were tested for bioburden and endotoxin levels (MicroSafe, Millipore, Leiden, The Netherlands) and have passed pre-set release criteria for animal experiments.

Mice were intramuscularly immunized in the quadriceps of the hind legs with 100 μl (50 μl per leg) containing various doses of Ad26.ZIKV.M-Env or Ad26.Empty under isoflurane anesthesia. ZIKV challenges were performed 4 weeks after immunization with 10^5^ vp (corresponding to 10^2^ PFU) ZIKV-BR (Brazil ZKV2015; Genbank KU497555.1) in 100 μL via the retro orbital intravenous route under isoflurane anesthesia.

NHP received a single intramuscular injection with 0.5 ml containing 10^11^ vp Ad26.ZIKV.M-Env or formulation buffer and were challenged 4 weeks later with 10^6^ vp (corresponding to 10^3^ PFU) of ZIKV-BR via the subcutaneous route.

### Env ELISA

Mouse or NHP IgG antibodies to ZIKV Env were measured by ELISA, using a commercially available kit (Alpha Diagnostics mouse, RV-403120-1; NHP, RV-403110-1). Serially diluted serum samples and controls were applied to 96-well Env pre-coated plates. ZIKV Env-specific antibodies were detected by HRP-labeled anti-mouse or anti-monkey IgG (30 min at RT) and 3,3',5,5'-Tetramethylbenzidine (TMB) substrate. The reaction was stopped, followed by optical density measurement at 450 nm. The titer was determined as the log10 value of first dilution above 2x background (mouse) or 5x the background (NHP) that was defined as the OD value of naïve serum. The lower limit of detection defined as one dilution step below the start dilution of the samples (0.92 on log10 scale).

Alternatively, Env-binding IgG responses in mice were determined by an in house developed ELISA that highly correlates to the commercially available Alpha Diagnostics mouse ELISA (S1 Fig). In brief, 96-well plates were coated overnight with ZIKV recombinant Env protein (Meridian Life Sciences, R01635). On the next day plates were washed and blocked for 1 h with PBS containing 2% bovine serum albumin (Sigma, A7030-10G). Serially diluted serum samples, controls and serial dilution of mAb ZV-67 (Absolute Antibody, AB00812-2.) as standard were added. Plates were incubated for 1 h at RT. After washing, ZIKV Env-specific mouse antibodies were detected using an HRP-labeled anti-mouse IgG (KPL, 474–182) (1 h at RT) followed by a washing step and luminescence readout with LumiGlow substrate (Sera Care, 5430–0041) for 30 minutes. The log10 relative potency of a sample was calculated as the log10 ratio of the IC50 of the sample over the standard on the same plate. The IC50 values of the samples and standard on a plate were derived from a 4-parameter logistic curve fit per sample or standard with a common upper and lower asymptote and a common slope across samples and standard. The reported Env-specific antibody titers were defined as the log10 of the relative potency multiplied by 1000.

### Viral loads by RT-PCR

ZIKV viral loads were determined by RT-PCR as previously described [8]. Briefly, RNA was extracted from serum/plasma or other body fluids with a QIAcube HT purification kit (Qiagen, Hilden, Germany). The viral RNA was converted to cDNA using Superscript III reverse transcriptase with reverse primers targeting ZIKV Capsid. cDNA was subjected to RT-PCR using a TaqMan assay directed to CAP. In each run, a standard series of CAP mRNA was included to quantify the RNA in the samples. Final viral loads were calculated as virus particles per mL serum by multiplying the titer as determined by the RT-PCR with the total of the dilution factors of the individual steps. The limit of detection was 100 copies per mL or per 10^6^ cells.

### ZIKV neutralization assay (FRNT)

Focus reduction neutralization tests (FRNT) were conducted at Southern Research (Frederick, MD, USA). Vero cells were seeded at a concentration of 2x10^4^ cells/well in 96-well plates 24 h prior to the assay initiation. Heat-inactivated serum samples were serially diluted prior to being mixed and incubated with input virus ZIKV-PR (strain PRVABC59) for 1 h at 37°C. Cell-seeded 96-well plates were infected with 100 μl of the virus/serum mixtures for 1 h before the addition of agarose containing overlay media. Each serum dilution was tested in triplicate wells. Approximately 24 h after infection, ZIKV foci were detected using an anti-flavivirus detection antibody (Millipore, MAB10216), a horseradish peroxidase (HRP)-conjugated secondary antibody (Seracare, 5220–0341), and TrueBlue™ peroxidase substrate (KPL, 71-00-65). ZIKV foci were visualized and counted using an ImmunoSpot analyzer. Neutralizing antibody titers are reported as the inverse of the serum dilution estimated to reduce the number of input virus by 50% (FRNT50).

### IFNγ ELISPOT

ZIKV-specific cellular immune responses in the spleen of mice or blood of NHP were assessed by IFN-γ ELISPOT assays. Freshly isolated mouse splenocytes or NHP PBMCs were stimulated overnight with a pool of 146 15-meric overlapping peptides (2μg/ml per peptide), derived from Envelope Protein E (JPT, Berlin, Germany, PM-ZIKV-E) of ZIKV, or a pool of 49 15-meric overlapping peptides derived from Membrane glycoprotein M (JPT, PM-ZIKV-M) of ZIKV, covering the full sequence diversity of ZIKV or medium as control. The numbers of spot-forming units (SFU) were determined using mouse IFNγ ELISpot kit (Millipore, Feltham, Middlesex, UK) or a human IFNγ ELISPOT assay kit (CTL, OH, USA, hIFNgp), according to the manufacturer’s instructions, and calculated to numbers of SFU per 10^6^ cells. Background levels of mouse responses were calculated as the 95% percentile of the spot forming units (SFU) observed in non-stimulated splenocytes. For NHP, SFU per 10^6^ non-stimulated PBMC’s was subtracted from specific responses of corresponding individual NHP. Specific responses that were below zero after background subtraction were set to zero.

### Intracellular cytokine staining (ICS)

Freshly isolated mouse splenocytes were stimulated with Env or M-derived peptide pools (JPT, PM-ZIKV-E and PM-ZIKV-M; 4μg/ml per peptide) or medium as negative control and were co-stimulated with hamster anti-mouse CD28 (BD bioscience, 553294) and rat anti-mouse CD49d (BD bioscience, 553153) for 1 h before Golgiplug (containing Brefeldin A; BD Biosciences, 51-2301KZ) was added. After ~12 hs incubation at 37°C, 10% CO_2_, LIVE/DEAD® Fixable Violet Dead Cell staining (Invitrogen, L34955) was added for dead cell exclusion. Non-specific binding of surface antibodies was blocked by purified rat anti-mouse CD16/CD32 (BD Biosciences, 553142) and followed by cell surface staining with fluorescently labeled antibodies: FITC hamster anti-mouse CD3e (553062), PE/CP Cy5.5 rat anti-mouse CD4 (550954), APC/Cy7 rat anti-mouse CD8a (557654), all from BD Bioscience. Cells were fixed with Cytofix/Cytoperm solution (BD Biosciences, 51-2090KZ) and permeabilized with BD Perm/Wash buffer (BD Biosciences, 51-2091KZ). Intracellular cytokines were stained with fluorescently labeled antibodies with PE rat anti-mouse IFNγ (554412), PE/Cy7 rat anti-mouse TNFα (557644) and APC rat anti-mouse IL-2 (554429), all from BD bioscience. The percentage of CD3^+^CD4^+^ and CD3^+^CD8^+^ T cells expressing IFNγ, IL-2, or TNFα was determined using BD FACS Canto™II (Becton Dickson B.V.) fluorescence-activated cell sorting (FACS) and analyzed using FlowJo software version 9.6.1 (Ashland, OR).

### Statistical analysis

Most mouse immunogenicity experiments presented in this manuscript were exploratory of nature and only post-hoc trend analysis of the dose dependency of the responses using the nonparametric Jonckheere-Terpstra test with the Ad26.empty group set as lowest dose was performed. For the comparison between prime and prime-boost regimens of Ad26.ZIKV.001 in C57BL/6 mice, Env-binding titers in serum and the different cellular readouts in the prime only regimen were compared to the responses in the prime-boost regimen in an across dose comparisons at the different sampling time points by ANOVA. In addition, to test whether antibody levels were maintained, trend analysis of repeated measurements in time was performed.

For the mouse efficacy studies, breakthrough rates of the Ad26.ZIKV.M-Env candidate with doses 4x10^7^, 2x10^8^ and 1x10^9^ were compared to the Ad26.Empty negative control group using a 2-sided Fisher’s exact test with two-fold Bonferroni adjustment, since a second vaccine candidate was included in the experiment that is out of scope of this manuscript, and using a stepwise approach starting with the highest dose per candidate. This stepwise approach was performed under the assumption that for each candidate the higher doses give lower breakthrough. For the area under the curve (AUC) viral load and Env-binding antibody responses the comparisons with the negative control group was done in the same way as for the breakthrough rate except that the Wilcoxon rank-sum test was used instead of Fisher’s exact test.

For the NHP challenge data the outcome of viral titer in plasma, CSF, urine and saliva measured by RT-qPCR during the challenge phase was summarized as AUC and compared between sham injected and Ad26.ZIKV.M-Env immunized NHPs with the Wilcoxon rank-sum test. Env- and NS1-binding and ZIKV neutralizing antibody responses induced by sham injection or Ad26.ZIKV.M-Env immunization were also compared using the Wilcoxon rank-sum test.

All statistical analyses were performed using SAS version 9.4 (SAS Institute Inc., USA) or R version 3.3.3. Statistical significance level was set at α = 0.05.

## Supporting information

S1 Checklist (DOCX)

S1 Fig **Humoral immune responses in Balb/c and SJL mice:** Env-specific binding IgG antibody titers (A and F) or ZIKV-PR neutralization titers (B) were determined in sera of Balb/c or SJL mice immunized with Ad26.ZIKV.M-Env (n = 4–5) or Ad26.Empty (n = 3) at the doses indicated, at 4 weeks post immunization. The Env-specific IgG titer was determined using a commercially available ELISA kit (Alpha Diagnostics) and expressed as the log10 of the inverse first dilution above 2x background values of naïve sera. Neutralizing antibody titers were measured by FRNT and are reported as the log10 of the inverse serum dilution that reduce the infectivity of input virus by 50% (IC50). The mean responses per group are indicated with a horizontal line. The dotted line shows the lower limit of detection. (C) The ratio (log10) of VNA and Env-binding titers. Only ratios were calculated when both VNA and Env-binding antibody responses were above limit of detection. ‘Not done’ (Nd) indicated that no ratio was calculated. (D and E) show correlations between Env-binding and ZIKV neutralizing antibody responses in Balb/c or SJL mice. (G) shows the correlation between Env-titers measured by the commercially available ELISA kit (Alpha Diagnostics) and the in-house developed Env-ELISA. Asterisks indicate statistically significant trend (*p<0.05, **p<0.01 and ***p<0.001) and “ns” indicates no statistical significant trend.
(DOCX)

S2 Fig A single immunization with Ad26.ZIKV.M-Env dose dependently induces ZIKV-specific CD4^+^ and CD8^+^ reactive T cells in C57BL/6 mice. Env and M-specific TNFα and IL2 responses were determined by ICS in splenocytes from C57BL/6 mice immunized with Ad26.ZIKV.M-Env (n = 5) or Ad26.Empty (n = 3) at the doses indicated, at 4 weeks post immunization. Splenocytes were stimulated overnight with Env-specific (A-D) or M specific (E-H) peptide pools. TNFα (A, B, E and F) or IL2 (C, D, G and H) was measured in CD3^+^CD4^+^ or CD3^+^CD8^+^ gated cells by ICS and FACS analysis and the percentage of CD3^+^CD4^+^ and CD3^+^CD8^+^ splenocytes producing TNFα or IL2 is depicted. The geometric mean response per group is indicated with a horizontal line. Asterisks indicate statistically significant trend (*p<0.05, **p<0.01 and ***p<0.001) and “ns” indicates no statistical significant trend.
(DOCX)

S3 Fig A single immunization with Ad26.ZIKV.M-Env dose dependently induces durable ZIKV-specific cellular responses in Balb/c and SJL mice. Env and M-specific IFNγ responses were determined by ELISPOT in splenocytes from Balb/c and SJL mice immunized with Ad26.ZIKV.M-Env (n = 3–5) or Ad26.Empty (n = 3) at the doses indicated, at 4 weeks post immunization. Splenocytes were stimulated overnight with Env-specific (A-B) or M specific (C-D) peptide pools. The number of IFNγ spot forming units (SFU) per 10^6^ splenocytes is shown. The geometric mean response per group is indicated with a horizontal line. The dotted lines indicate the background of the assays. Asterisks indicate statistically significant trend (*p<0.05, **p<0.01 and ***p<0.001) and “ns” indicates no statistical significant trend.
(DOCX)

S4 Fig Env-specific binding antibody responses do not increase after boost immunization. Env-specific binding IgG antibody titers (A-C) were determined in sera of C57BL/6 mice prime or prime boost immunized with Ad26.ZIKV.M-Env (n = 5) at the doses indicated, at 4 (pre-boost), 8 and 10 weeks after prime immunization by using an in-house developed ELISA assay. Dots represent individual responses of the relative potency (log10) compared to a ZIKV Env-specific monoclonal antibody ZV-67, and the mean per group is indicated with a horizontal line. Blue (prime only) and green (prime-boost). “ns” indicated non-statistical significance comparing prime versus prime-boost in an across dose analysis.
(DOCX)

S5 Fig Ad26.ZIKV.M-Env confers protection in NHP as measured by viral loads in CSF, Urine and saliva. (A-F) Protective efficacy against viremia was determined in NHP after subcutaneous challenge with 10^3^ pfu ZIKV- 4 weeks post-immunization. Viral load was determined by RT-PCR in CSF (A-B), Urine (C-D) and saliva (E-F) obtained pre-challenge and at day 3 and 7 after challenge and were depicted as log10 ZIKV copies/mL plasma.
(DOCX)

S1 Table **A**: Draize scores were determined before vaccination (day 0 + 0h), 6h after vaccine administration (day 0 + 6h) and daily for 3 consecutive days post immunization according to the scoring system (S1B Table). In case any reaction was noted at day 3, scoring continued until reaction completely resolved. ‘–‘ indicates that no scoring was performed. **B: Overview methodology draize scoring.**
(DOCX)

S2 Table Body temperature in degrees Fahrenheit (°F). Green indicates relative low and red indicates relative high temperature of an individual animal. Vaccine or sham was administrated at day 0 (+0 h).
(DOCX)
