# Supplementary material for: Adenoviral vector type 26 encoding Zika virus (ZIKV) M-Env antigen induces humoral and cellular immune responses and protects mice and nonhuman primates against ZIKV challenge
Source: PLoS One. 2018 Aug 24;13(8):e0202820. doi: 10.1371/journal.pone.0202820 (PMC6108497; doi:10.1371/journal.pone.0202820)

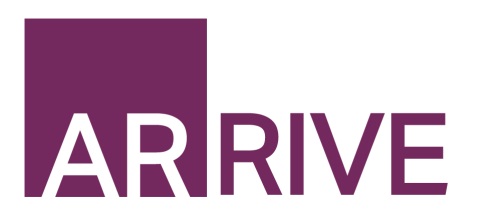


The ARRIVE Guidelines Checklist

Animal Research: Reporting In Vivo Experiments

Carol Kilkenny^1^, William J Browne^2^, Innes C Cuthill^3^, Michael Emerson^4^ and Douglas G Altman^5^

*^1^The National Centre for the Replacement, Refinement and Reduction of Animals in Research, London, UK, ^2^School of Veterinary Science, University of Bristol, Bristol, UK, ^3^School of Biological Sciences, University of Bristol, Bristol, UK, ^4^National Heart and Lung Institute, Imperial College London, UK, ^5^Centre for Statistics in Medicine, University of Oxford, Oxford, UK.*

|  | | ITEM | RECOMMENDATION | | Section/ Paragraph | | |
| --- | --- | --- | --- | --- | --- | --- | --- |
| 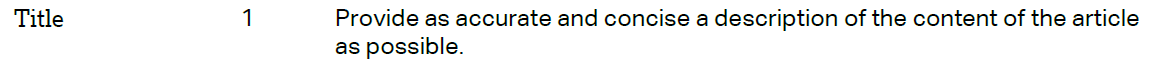 | | | | See Title | | |  |
| 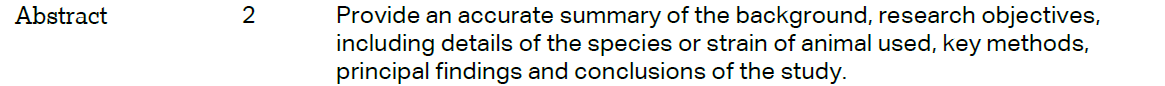 | | | | See Abstract | | |  |
| INTRODUCTION | | | |  | | |  |
| 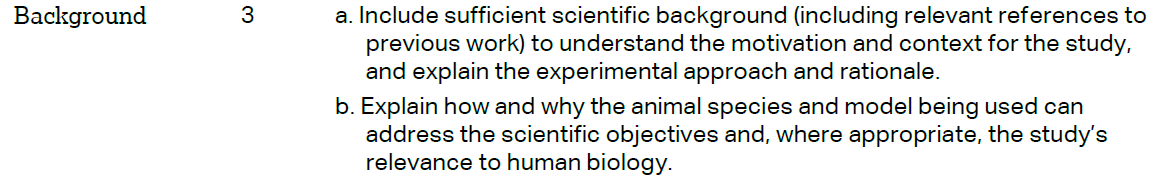 | | | | See Introduction | | |  |
| 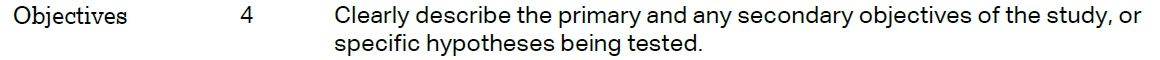 | | | | See Introduction | | |  |
| METHODS | | | |  | | |  |
| 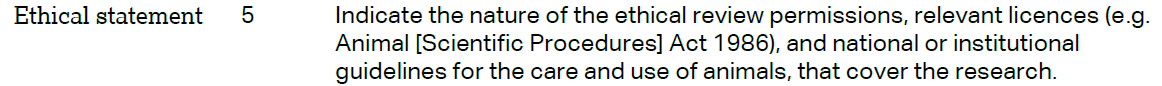 | | | | See Ethical statement (Materials and methods section) | | |  |
| 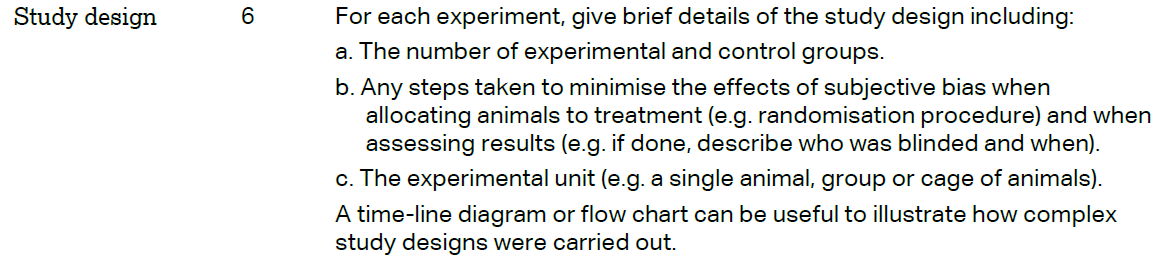 | | | | See Vaccine, animals and challenge (Materials and methods section) | | |  |
| 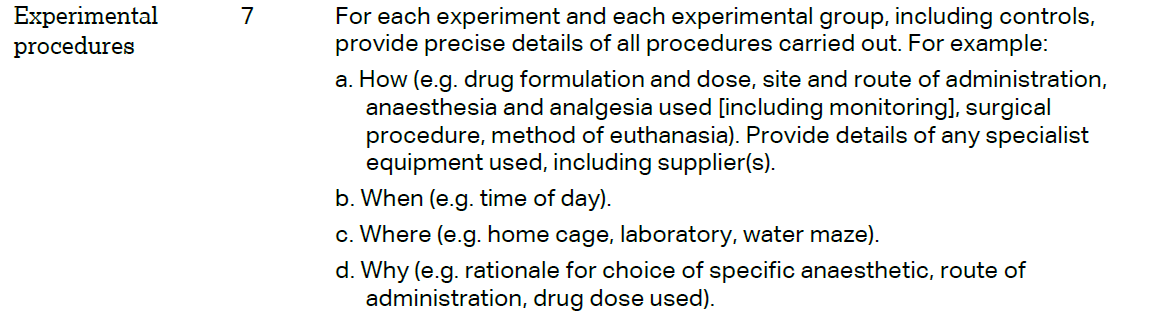 | | | | See Vaccine, animals and challenge (Materials and methods section) | | |  |
| 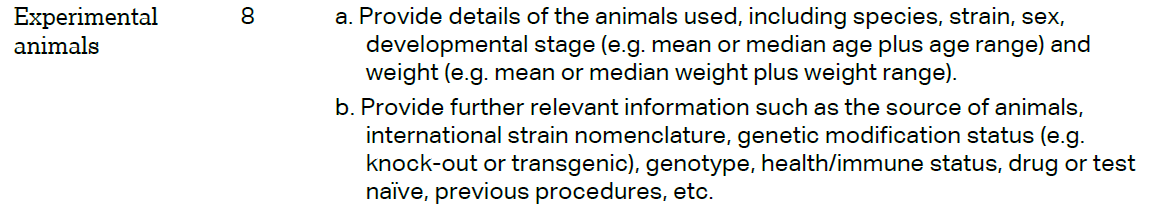 | | | | See Vaccine, animals and challenge (Materials and methods section) | | |  |
| 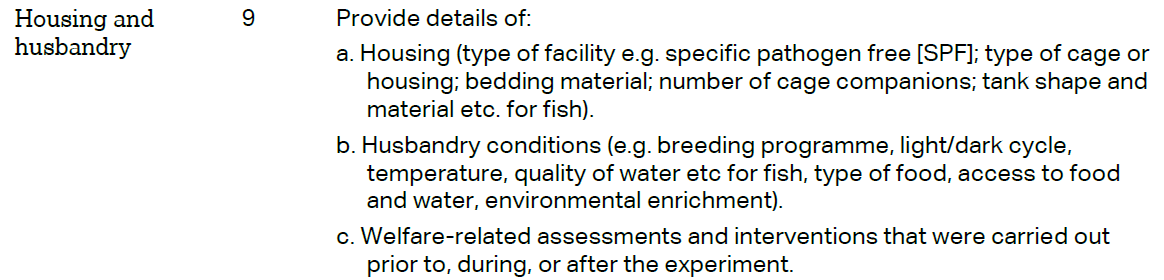 | | | See Vaccine, animals and challenge (Materials and methods section) | | |  |  |
| 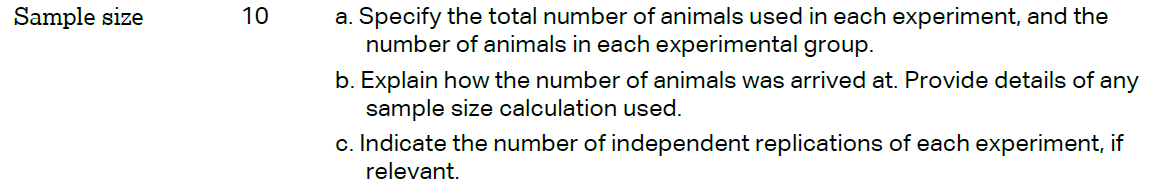 | | | Number of animals per experiment are mentioned in the Figure captions | | |  |  |
| 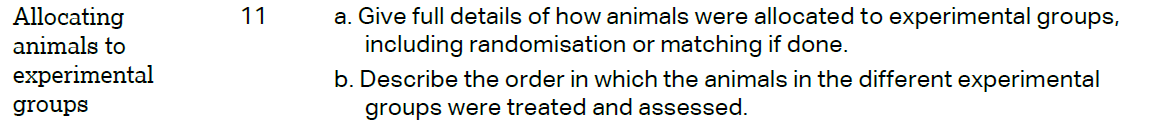 | | | Animals were randomly distributed into the different treatment groups. All immunological and virological readouts were performed blinded. | | |  |  |
| 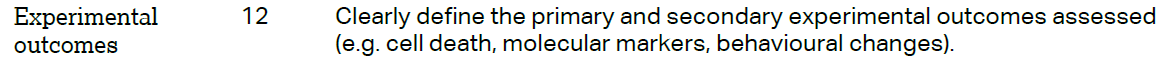 | | | Immunogenicity readouts in serum by ELISA or VNA (FRNT) and or in splenocytes/blood by ELISPOT or ICS and FACS analysis. Virology readout in plasma/serum, CSF Urine and saliva. | | |  |  |
| 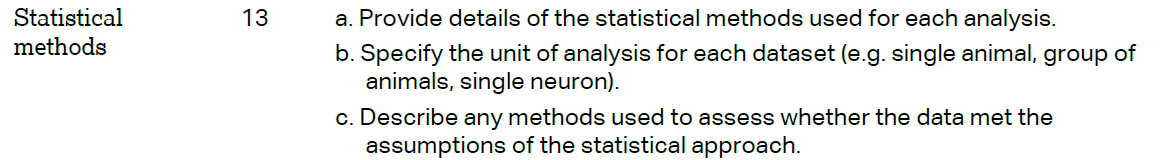 | | | See Statistical analysis (Materials and methods section) | | |  |  |
| RESULTS | | |  | | |  |  |
| 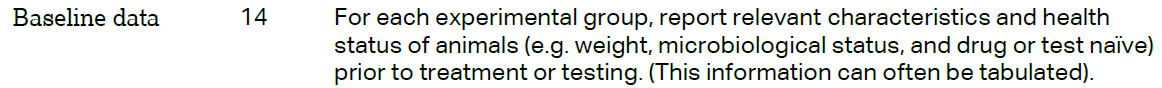 | | | See Vaccine, animals and challenge (Materials and methods section) | | |  |  |
| 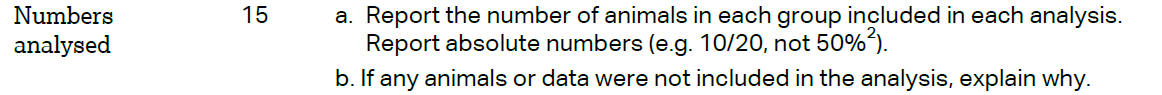 | | | No animals were excluded for analysis except for 3 animals that were excluded from the ELISPOT analysis (Figure 2 A-C) due to high variation between the duplicates measurements (in accordance to our pre-set criteria; SOP) | | |  |  |
| 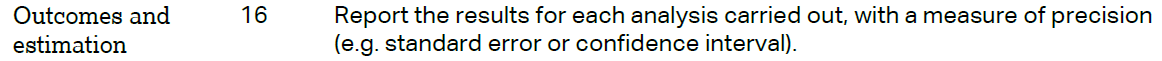 | | | Individual responses our mean/geomean of group responses including SD are displayed in all figures. | | |  |  |
| 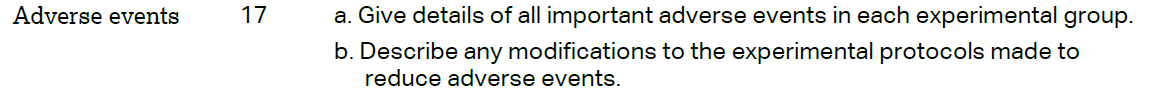 | | | See supplementary table 1 and 2 | | |  |  |
| DISCUSSION | | |  | | |  |  |
| 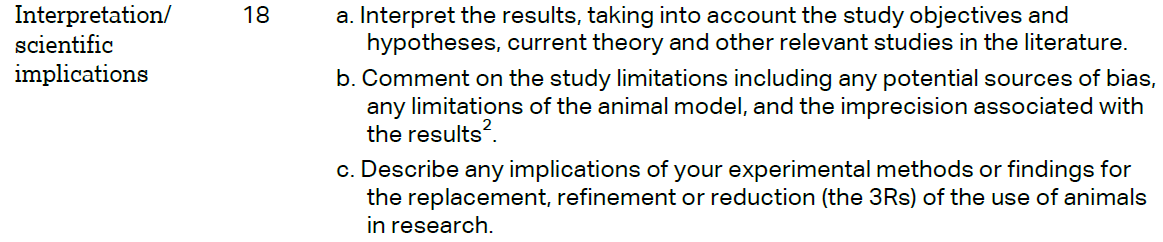 | | | See Discussion section | | |  |  |
| 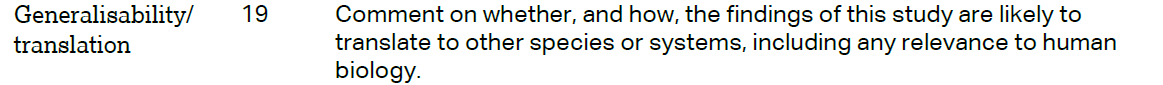 | | | The data presented in the manuscript has led to the clinical testing of the vaccine candidate | | |  |  |
| 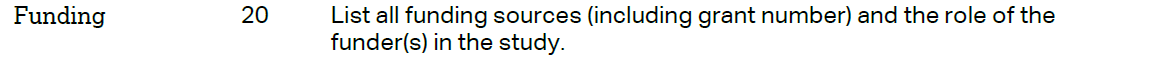 | | | | See the Funding Statement and Competing Interests Statement | |  |  |


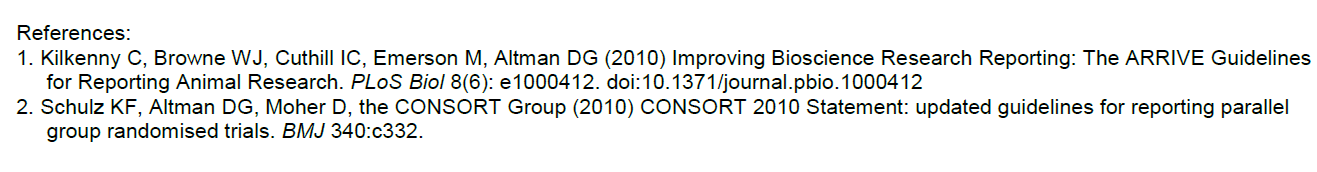

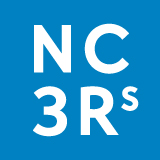

Supplement: S1 Checklist — (DOCX) [file pone.0202820.s001.docx]
