# Supplementary material for: Adenoviral vector type 26 encoding Zika virus (ZIKV) M-Env antigen induces humoral and cellular immune responses and protects mice and nonhuman primates against ZIKV challenge
Source: PLoS One. 2018 Aug 24;13(8):e0202820. doi: 10.1371/journal.pone.0202820 (PMC6108497; doi:10.1371/journal.pone.0202820)
Supplement: S1 Fig — Humoral immune responses in Balb/c and SJL mice: Env-specific binding IgG antibody titers (A and F) or ZIKV-PR neutralization titers (B) were determined in sera of Balb/c or SJL mice immunized with Ad26.ZIKV.M-Env (n = 4–5) or Ad26.Empty (n = 3) at the doses indicated, at 4 weeks post immunization. The Env-specific IgG titer was determined using a commercially available ELISA kit (Alpha Diagnostics) and expressed as the log10 of the inverse first dilution above 2x background values of naïve sera. Neutralizing antibody titers were measured by FRNT and are reported as the log10 of the inverse serum dilution that reduce the infectivity of input virus by 50% (IC50). The mean responses per group are indicated with a horizontal line. The dotted line shows the lower limit of detection. (C) The ratio (log10) of VNA and Env-binding titers. Only ratios were calculated when both VNA and Env-binding antibody responses were above limit of detection. ‘Not done’ (Nd) indicated that no ratio was calculated. (D and E) show correlations between Env-binding and ZIKV neutralizing antibody responses in Balb/c or SJL mice. (G) shows the correlation between Env-titers measured by the commercially available ELISA kit (Alpha Diagnostics) and the in-house developed Env-ELISA. Asterisks indicate statistically significant trend (*p<0.05, **p<0.01 and ***p<0.001) and “ns” indicates no statistical significant trend. (DOCX) [file pone.0202820.s002.docx]

**S1 Fig: Humoral immune responses in Balb/c and SJL mice:** Env-specific binding IgG antibody titers (A and F) or ZIKV-PR neutralization titers (B) were determined in sera of Balb/c or SJL mice immunized with Ad26.ZIKV.M-Env (n=4-5) or Ad26.Empty (n=3) at the doses indicated, at 4 weeks post immunization. The Env-specific IgG titer was determined using a commercially available ELISA kit (Alpha Diagnostics) and expressed as the log10 of the inverse first dilution above 2x background values of naïve sera. Neutralizing antibody titers were measured by FRNT and are reported as the log10 of the inverse serum dilution that reduce the infectivity of input virus by 50% (IC50). The mean responses per group are indicated with a horizontal line. The dotted line shows the lower limit of detection. (C) The ratio (log10) of VNA and Env-binding titers. Only ratios were calculated when both VNA and Env-binding antibody responses were above limit of detection. ‘Not done’ (Nd) indicated that no ratio was calculated. (D and E) show correlations between Env-binding and ZIKV neutralizing antibody responses in Balb/c or SJL mice. (G) shows the correlation between Env-titers measured by the commercially available ELISA kit (Alpha Diagnostics) and the in-house developed Env-ELISA. Asterisks indicate statistically significant trend (*p<0.05, **p<0.01 and ***p<0.001) and “ns” indicates no statistical significant trend.
