# Supplementary material for: Adenoviral vector type 26 encoding Zika virus (ZIKV) M-Env antigen induces humoral and cellular immune responses and protects mice and nonhuman primates against ZIKV challenge
Source: PLoS One. 2018 Aug 24;13(8):e0202820. doi: 10.1371/journal.pone.0202820 (PMC6108497; doi:10.1371/journal.pone.0202820)
Supplement: S2 Fig — Env and M-specific TNFα and IL2 responses were determined by ICS in splenocytes from C57BL/6 mice immunized with Ad26.ZIKV.M-Env (n = 5) or Ad26.Empty (n = 3) at the doses indicated, at 4 weeks post immunization. Splenocytes were stimulated overnight with Env-specific (A-D) or M specific (E-H) peptide pools. TNFα (A, B, E and F) or IL2 (C, D, G and H) was measured in CD3+CD4+ or CD3+CD8+ gated cells by ICS and FACS analysis and the percentage of CD3+CD4+ and CD3+CD8+ splenocytes producing TNFα or IL2 is depicted. The geometric mean response per group is indicated with a horizontal line. Asterisks indicate statistically significant trend (*p<0.05, **p<0.01 and ***p<0.001) and “ns” indicates no statistical significant trend. (DOCX) [file pone.0202820.s003.docx]

**S2 Fig: A single immunization with Ad26.ZIKV.M-Env dose dependently induces ZIKV-specific CD4^+^ and CD8^+^ reactive T cells in C57BL/6 mice.** Env and M-specific TNFα and IL2 responses were determined by ICS in splenocytes from C57BL/6 mice immunized with Ad26.ZIKV.M-Env (n=5) or Ad26.Empty (n=3) at the doses indicated, at 4 weeks post immunization. Splenocytes were stimulated overnight with Env-specific (A-D) or M specific (E-H) peptide pools. TNFα (A, B, E and F) or IL2 (C, D, G and H) was measured in CD3^+^CD4^+^ or CD3^+^CD8^+^ gated cells by ICS and FACS analysis and the percentage of CD3^+^CD4^+^ and CD3^+^CD8^+^ splenocytes producing TNFα or IL2 is depicted. The geometric mean response per group is indicated with a horizontal line. Asterisks indicate statistically significant trend (*p<0.05, **p<0.01 and ***p<0.001) and “ns” indicates no statistical significant trend.
