# Supplementary material for: Adenoviral vector type 26 encoding Zika virus (ZIKV) M-Env antigen induces humoral and cellular immune responses and protects mice and nonhuman primates against ZIKV challenge
Source: PLoS One. 2018 Aug 24;13(8):e0202820. doi: 10.1371/journal.pone.0202820 (PMC6108497; doi:10.1371/journal.pone.0202820)
Supplement: S3 Fig — Env and M-specific IFNγ responses were determined by ELISPOT in splenocytes from Balb/c and SJL mice immunized with Ad26.ZIKV.M-Env (n = 3–5) or Ad26.Empty (n = 3) at the doses indicated, at 4 weeks post immunization. Splenocytes were stimulated overnight with Env-specific (A-B) or M specific (C-D) peptide pools. The number of IFNγ spot forming units (SFU) per 106 splenocytes is shown. The geometric mean response per group is indicated with a horizontal line. The dotted lines indicate the background of the assays. Asterisks indicate statistically significant trend (*p<0.05, **p<0.01 and ***p<0.001) and “ns” indicates no statistical significant trend. (DOCX) [file pone.0202820.s004.docx]

**S3 Fig: A single immunization with Ad26.ZIKV.M-Env dose dependently induces durable ZIKV-specific cellular responses in Balb/c and SJL mice.** Env and M-specific IFNγ responses were determined by ELISPOT in splenocytes from Balb/c and SJL mice immunized with Ad26.ZIKV.M-Env (n=3-5) or Ad26.Empty (n=3) at the doses indicated, at 4 weeks post immunization. Splenocytes were stimulated overnight with Env-specific (A-B) or M specific (C-D) peptide pools. The number of IFNγ spot forming units (SFU) per 10^6^ splenocytes is shown. The geometric mean response per group is indicated with a horizontal line. The dotted lines indicate the background of the assays. Asterisks indicate statistically significant trend (*p<0.05, **p<0.01 and ***p<0.001) and “ns” indicates no statistical significant trend.
