# Supplementary material for: Adenoviral vector type 26 encoding Zika virus (ZIKV) M-Env antigen induces humoral and cellular immune responses and protects mice and nonhuman primates against ZIKV challenge
Source: PLoS One. 2018 Aug 24;13(8):e0202820. doi: 10.1371/journal.pone.0202820 (PMC6108497; doi:10.1371/journal.pone.0202820)
Supplement: S4 Fig — Env-specific binding IgG antibody titers (A-C) were determined in sera of C57BL/6 mice prime or prime boost immunized with Ad26.ZIKV.M-Env (n = 5) at the doses indicated, at 4 (pre-boost), 8 and 10 weeks after prime immunization by using an in-house developed ELISA assay. Dots represent individual responses of the relative potency (log10) compared to a ZIKV Env-specific monoclonal antibody ZV-67, and the mean per group is indicated with a horizontal line. Blue (prime only) and green (prime-boost). “ns” indicated non-statistical significance comparing prime versus prime-boost in an across dose analysis. (DOCX) [file pone.0202820.s005.docx]

**S4 Fig: Env-specific binding antibody responses do not increase after boost immunization**. Env-specific binding IgG antibody titers (A-C) were determined in sera of C57BL/6 mice prime or prime boost immunized with Ad26.ZIKV.M-Env (n=5) at the doses indicated, at 4 (pre-boost), 8 and 10 weeks after prime immunization by using an in-house developed ELISA assay. Dots represent individual responses of the relative potency (log10) compared to a ZIKV Env-specific monoclonal antibody ZV-67, and the mean per group is indicated with a horizontal line. Blue (prime only) and green (prime-boost). “ns” indicated non-statistical significance comparing prime versus prime-boost in an across dose analysis.
