# Supplementary material for: Adenoviral vector type 26 encoding Zika virus (ZIKV) M-Env antigen induces humoral and cellular immune responses and protects mice and nonhuman primates against ZIKV challenge
Source: PLoS One. 2018 Aug 24;13(8):e0202820. doi: 10.1371/journal.pone.0202820 (PMC6108497; doi:10.1371/journal.pone.0202820)
Supplement: S5 Fig — (A-F) Protective efficacy against viremia was determined in NHP after subcutaneous challenge with 103 pfu ZIKV- 4 weeks post-immunization. Viral load was determined by RT-PCR in CSF (A-B), Urine (C-D) and saliva (E-F) obtained pre-challenge and at day 3 and 7 after challenge and were depicted as log10 ZIKV copies/mL plasma. (DOCX) [file pone.0202820.s006.docx]

**S5 Fig: Ad26.ZIKV.M-Env confers protection in NHP as measured by viral loads in CSF, Urine and saliva.** (A-F) Protective efficacy against viremia was determined in NHP after subcutaneous challenge with 10^3^ pfu ZIKV- 4 weeks post-immunization. Viral load was determined by RT-PCR in CSF (A-B), Urine (C-D) and saliva (E-F) obtained pre-challenge and at day 3 and 7 after challenge and were depicted as log10 ZIKV copies/mL plasma.
