# Supplementary material for: Adenoviral vector type 26 encoding Zika virus (ZIKV) M-Env antigen induces humoral and cellular immune responses and protects mice and nonhuman primates against ZIKV challenge
Source: PLoS One. 2018 Aug 24;13(8):e0202820. doi: 10.1371/journal.pone.0202820 (PMC6108497; doi:10.1371/journal.pone.0202820)
Supplement: S1 Table — A: Draize scores were determined before vaccination (day 0 + 0h), 6h after vaccine administration (day 0 + 6h) and daily for 3 consecutive days post immunization according to the scoring system (S1B Table). In case any reaction was noted at day 3, scoring continued until reaction completely resolved. ‘–‘ indicates that no scoring was performed. B: Overview methodology draize scoring. (DOCX) [file pone.0202820.s007.docx]

**S1 Table A**: Draize scores were determined before vaccination (day 0 + 0h), 6h after vaccine administration (day 0 + 6h) and daily for 3 consecutive days post immunization according to the scoring system (Sup Table 1B). In case any reaction was noted at day 3, scoring continued until reaction completely resolved. ‘–‘ indicates that no scoring was performed.

**S1 Table B: Overview methodology draize scoring**
