# Supplementary material for: Adenoviral vector type 26 encoding Zika virus (ZIKV) M-Env antigen induces humoral and cellular immune responses and protects mice and nonhuman primates against ZIKV challenge
Source: PLoS One. 2018 Aug 24;13(8):e0202820. doi: 10.1371/journal.pone.0202820 (PMC6108497; doi:10.1371/journal.pone.0202820)
Supplement: S2 Table — Green indicates relative low and red indicates relative high temperature of an individual animal. Vaccine or sham was administrated at day 0 (+0 h). (DOCX) [file pone.0202820.s008.docx]

**S2 Table:** Body temperature in degrees Fahrenheit (°F). Green indicates relative low and red indicates relative high temperature of an individual animal. Vaccine or sham was administrated at day 0 (+0 h)
